# S239. PREDICTIVE ACCURACY FOR WORK OUTCOME IN PATIENTS WITH SCHIZOPHRENIA

**DOI:** 10.1093/schbul/sby018.1026

**Published:** 2018-04-01

**Authors:** Chika Sumiyoshi, Haruo Fujino, Tomiki Sumiyoshi, Hidenaga Yamamori, Noriko Kudo, Hirotsugu Azechi, Michiko Fujimoto, Yuka Yasuda, Kazutaka Ohi, Ryota Hashimoto

**Affiliations:** 1 Fukushima University; 2 Oita University; 3 Translational Medical Center, National Center of Neurology and Psychiatry; 4 Osaka University Graduate School of Medicine; 5 Osaka University; 6 Kanazawa Medical University

## Abstract

**Background:**

Functional capacity (i.e., what a person can do under optimal conditions) may not be directly transferred to functional performance (i.e., what a person actually does in a real-world situation) in patients with schizophrenia due to internal or external variables (Bowie et al., 2006). This discrepancy would be larger for relatively high functioning patients compared to those with low functional status, because of a greater likelihood of being exposed to real-world settings in the former group. The purpose of this study was to determine whether prediction of work outcomes would become less reliable in patients who work for long hours than those who do not.

**Methods:**

Subjects: One-hundred and thirty-seven Japanese patients meeting DSM-IV-TR criteria for schizophrenia and 156 healthy adults entered the study. The study was approved by the Ethics Committee of Osaka University.

Assessment: Current and premorbid intelligence (WAIS-3 and Japanese version of the Adult Reading Test) and functional outcomes (the UCSD Performance-Based Skills Assessment-Brief and the Social Functioning Scale Individuals’ version Modified for MATRICS-PASS) were assessed. The Positive and Negative Syndrome Scales (PANSS) and the Schizophrenia Quality of Life Scale Japanese version were also for patients. Total work hours per week, obtained from the Social Activity Assessment, was used as a measure of work outcome

Analyses: Four separate multiple logistic regression analyses were conducted to predict work outcomes. Independent variables were found to be significant by means of the group comparisons. Dependent variable, i.e., the number of work hours, was dichotomized by a criterion of 0, 10, 20, or 30 hours/week. At each level, patients were classified into either the above (=1) or the below (=0) criterion (observed outcomes). Estimated probabilities were calculated using factors that remained significant in regression models (intelligence decline, psychiatric symptoms, and social function). Predictive accuracy was calculated by summing the ratios correctly classified (i.e., a patient’s observed outcome=1[0] and the estimated probability > 0.5 [< 0.5]). In addition, frequency distributions for observed outcomes were generated on the axis of estimated probabilities to examine the characteristics of correct classification.

**Results:**

Overall, estimation was more accurate at higher criteria, yielding 80–87% accuracy at 20 or 30 hours/week criteria (c.f., 67–70% at 0 or 10 hours/week criteria). Distributions for the observed outcomes=0 had the peaks at the part of the estimate probabilities < 0.5, while those for observed outcomes=1 were relatively uniform, lacking the peaks at the part of estimated probabilities >0.5. These results indicate that the classification was less accurate in patients who exceeded the criteria (observed outcomes=1) compared to those who did not (observed outcomes=0).

**Discussion:**

The current study showed that prediction for work outcome was less reliable in patients who attained relatively better work outcome. One of the reasons would be sextrinsic variables, such as local economy, welfare services, and stigma, may intervene between functional capacity and work outcome (Buchanan et al., 2005). Also, some of high functioning patients may have better insight into their work capacity (Gould et al., 2013), which may refrain them from engaging in long-hour work.

**References:**

1. Bowie CR et al. Am J Psychiatry. 2006;163:418–425.

2. Buchanan RW et al. Schizophr Bull. 2005;31:5–19.

3. Gould F, et al. Psychiatry Res. 2013;207:19-2.

